# Holy Communion and Infection Transmission: A Literature Review

**DOI:** 10.7759/cureus.8741

**Published:** 2020-06-21

**Authors:** Dimitrios Anyfantakis

**Affiliations:** 1 Primary Care, Primary Health Care Centre of Kissamos, Chania, GRC

**Keywords:** religion, holly communion, infection, coronavirus, health public, eucharist

## Abstract

The Holy Communion originated in the Last Supper of Jesus Christ, nearly 2,000 years ago. According to the Bible, the night before his crucifixion, Jesus Christ shared with his 12 apostles a meal of bread and wine. During the meal, Christ instructed his disciplines to eat and drink in his memory, saying that bread is his body and wine is his blood. Today, faithful people worldwide share the consecrated bread and wine retracted from a chalice with a Holy Communion spoon.

The novel coronavirus that emerged in December 2019 recorded a rapid exponential spread across space and time. The ongoing pandemic of coronavirus disease has affected people from all cultures and religions. In Greece, the pandemic concurred with the Easter celebration.

Measures of social distancing have been implemented. Among others, churches have closed their doors to the public in order to avoid religious mass gatherings. The issue of the novel coronavirus transmission by partaking Holy Communion has received much criticism. In this review, we aimed to retrieve articles that summarize the current knowledge on the selected topic. In order to offer a balanced analysis of the subject, we have also assessed the theological framework of the Holy mystery.

## Introduction and background

Eucharist or Holy Communion is a Christian ritual through which a member of the Church (bishop or presbyter) offers to the worshipers the Holy gifts, the consecrated bread and wine [1]. The word “Eucharist” derives from the Greek word “eucharistia” which means "thanksgiving". The Eucharistic practice in the Orthodox faith consists of dipping the consecrated bread into the chalice together with the consecrated wine [1]. This practice is named intinction [1]. The elements of the Holy Communion are retrieved from the chalice with a small spoon and are placed directly into the recipient’s mouth. Participants share Holy Communion from the same cup. Furthermore, the common spoon is not wiped between recipients [1].

A recent pandemic has recorded a rapid spread worldwide and has resulted in high rates of hospitalization and intensive care unit admission [2]. Social distancing policies have been implemented in order to avoid public gathering. In front of the Easter celebration in Greece, religious services have been performed behind closed doors for the public [2].

The issue of coronavirus transmission by sharing the Holy Communion has been a subject of debate between science and the Greek Orthodox Church. Currently, the German government, although permitted attendance on religious services, prohibited the participation of the faithful population in the mystery of Holy Communion.

In this paper, we aimed through a literature search of published studies to provide a comprehensive overview of this topic. In order to offer a balanced analysis, we also assessed theological and historical views.

## Review

Theological framework of the mystery of Holy Communion

The mystery of Eucharist, also called Holy Communion has been instituted by Jesus Christ before his death [3]. During the Passover meal, which is named Last Supper, Jesus gave his disciples bread and wine and told them: “Take it; this is my body. Then he took the cup, gave thanks, and gave it to them, saying, “Drink from it all of you; this is my blood” [4,5].

Previously he had stated that “Whoever eats my flesh and drinks my blood has eternal life, and I will raise them up at the last day” [4-6]. By saying this word, he wanted to confirm that the elements of the Holy Communion were his “body” and his “blood” and not simply bread and wine [4-6]. For over 200 years, the celebration of the Holy Communion makes people actual members of the Orthodox Church [4-6].

In the same direction, Saint John of Damascus clarifies that the consecrated bread and wine are not symbols of the body and blood of Christ, but Christ’s body itself [7]. He also underlines that the communion is realistic and not metaphoric, and by partaking Holy Communion human bodies become bodies of Jesus Christ [7].

According to Saint Ignatius, the Holy Eucharist, “is the medicine of immortality and the antidote against death, so that we might live forever in Jesus Christ” [8].

Therefore, Christian theology cannot accept that contact with the chalice or the Holy Communion spoon, may act as a vehicle of transmission of pathogens to the worshiper [3,6].

In support of this, priests consume the remainder content of the chalice at the end of Divine Liturgy. Consequently, they should be the first infected persons [9]. The case of the priest of the lepers on Spinalonga, Monk Chrysanthos Koutsoulogiannakis may sustain this argument [10]. Spinalonga is an islet located in north-eastern Crete that was historically used to isolate patients with Hansen’s disease from the healthy population (1903-1957) [10]. For a period of 10 years, Monk Chrysanthos served Holy Communion to the patients with Hansen’s disease. He partook of the Holy Communion from the same spoon, without getting infected. Similarly, Elder Evmenios Saridakis served and blessed patients in a Leprosy hospital on Saint Barbara, Athens, without manifesting the disease.

Scientific evidence on the transmission of infections by partaking in the Holy Communion

The issue of the potential transmission of infectious diseases through the Holy Communion has given rise to a growing number of research efforts since the late 19th century [11]. A hypothesis that pathogens of the mouth may contaminate wine on the communion cup has been formulated by Hobbs et al. [11].

Researchers have performed experiments, through which volunteers were asked to drink sacramental wine that contained 14.5% of alcohol from a common silver communion cup or chalice [11]. Remarkably, the number of pathogens located in the rim of the chalice was found considerably low [11]. The authors concluded that the risk of the transmission of the infection through a common communion cup is negligible [11]. Furthermore, rotation of the chalice was ineffective in reducing bacterial colonization [11]. Wiping the rim of the chalice with a cloth reduced bacterial counts by 90% [11].

In the same direction, Burrows and Hemmens investigated the potential transmission of pathogens, from one person to another by the common use of the chalice [12]. Interestingly, the authors have reported that under the most favorable conditions only 0.001% of organisms were transmitted from the saliva of one person to the mouth of another [12]. Remarkably, Streptococcus Pyogenes swabbed from the polished surface of the chalice died of rapidly [12].

Manangan et al. disclosed that the issue of potential transmission of bacteria through the common communion cup is controversial [13]. They stated that even if transmission occurs, it does not imply inoculation neither infection [13]. Disease requires a minimum number of pathogens to be transmitted from person to person [13]. Furthermore, the common communion cup has never been associated with a pandemic outbreak [13].

Despite the considerable debate on this issue, in 1998, the Centre of Disease and Control Prevention attempted to achieve a balance between scientific principles and respect for religious beliefs [13]. In this route, a study performed among 681 worshippers partaking Holy Communion disclosed that they did not exert a higher risk of infection compared to those with less or no religious service attendance [13].

Another study investigated the risk of contamination by partaking Holy Communion following the practice of intinction of the Holy gifts [14]. The authors reported that although intinction did not eliminate the risk, significantly reduced the hazard of infection compared to the practice of sipping from a common communion cup. They suggested intinction to be a safer alternative method for receiving the Holy Communion [14].

Fiedler et al. remarked the high risk that poses immunodeficient patients during Holy Communion and suggested the use of individual chalices for all the participants. They reported that intinction would be a more favorable method to avoid infections [15].

Expert medical opinions in regards to the transmission of coronavirus through participation in the mystery of Eucharist divided the scientific community in Greece [16]. Dr Eleni Giamarelou, a prominent Professor of Internal Medicine in the University of Athens, an expert in the area of infectious diseases, stated that "Holy Communion is the greatest mystery of the Orthodox faith that cannot be interpreted through logical reasoning" [16]. She also added that those who believe that through the Holy Communion receive the "body and the blood of Jesus Christ" and not simply wine and bread can partake without fearing the coronavirus [16]. She also argued against the use of personal plastic teaspoons [16]. Her opinion has received much criticism from the Greek politics and expert scientists. Metropolitan Mesogaias Nikolaos has disclosed an interesting statement [17]. The primary goal of science is the discovery of the truth of the created world [17]. Religion’s aim is the disclosure of the truths of God [17]. These will not be achieved if science is dominated by arrogance and religious thoughts by narrowness [17]. He emphasized that interpretation of the Holy Eucharist as a vehicle through which a contagious disease may be transmitted, derives from the lack of faith and the human rationality [17]. It is remarkable that in front of this human disaster, the requirement of a spiritual way of living has emerged. Medical doctors occupied in countries seriously affected by the coronavirus pandemic such as Italy, witnessed religious conversions among infected healthcare workers [18]. They have recognized the importance of spirituality and faith to alleviate stress and psychical sufferance [18].

A growing body of research efforts reports a beneficial effect of religiosity on immune functioning and mental health [19]. Faith and trust in the plan of God have been correlated with positive emotions such as hope, optimism, happiness, lower depression and a higher sense of internal control [20,21]. Interestingly, in the Orthodox Christian faith, positive emotions are considered the "fruits of the Holy spirit" (love, peace, forbearance, kindness, goodness, faithfulness, gentleness and self-control) [20]. Furthermore, spiritual health is strongly associated with somatic health, alcohol and smoking avoidance, physical activity [20,21].

## Conclusions

The potential transmission of coronavirus by partaking Holy Communion has divided Greek society, politics and medical experts. From the part of the science, the common communion cup may serve as a potential vehicle for transmission. However, the risk is considerably lower compared to other conditions of social gathering. Furthermore, the transmission of any infectious disease has never been documented. Definite answers cannot be obtained in this issue. Appropriate assessment of the Holy Eucharist needs to be extended beyond a partial scientific analysis. Science seems to stand in opposition with the concept of Holy Communion. The greatest "medicine of the soul and the body" cannot be explained with human reasoning and pure logical criteria. Furthermore, a balance between scientific views and respect for the spiritual needs of the believers is required. Living with spirituality and prayer relieves stress and suffering.
